# Chemical Synthesis, Proper Folding, Na_v_ Channel Selectivity Profile and Analgesic Properties of the Spider Peptide Phlotoxin 1

**DOI:** 10.3390/toxins11060367

**Published:** 2019-06-21

**Authors:** Sébastien Nicolas, Claude Zoukimian, Frank Bosmans, Jérôme Montnach, Sylvie Diochot, Eva Cuypers, Stephan De Waard, Rémy Béroud, Dietrich Mebs, David Craik, Didier Boturyn, Michel Lazdunski, Jan Tytgat, Michel De Waard

**Affiliations:** 1 Institut du Thorax, Inserm UMR 1087/CNRS UMR 6291, LabEx “Ion Channels, Science & Therapeutics”, F-44007 Nantes, France; sebastien.nicolas@univ-nantes.fr (S.N.); jerome.montnach@univ-nantes.fr (J.M.); stephan.de-waard@etu.univ-nantes.fr (S.D.W.); 2 Smartox Biotechnology, 6 rue des Platanes, F-38120 Saint-Egrève, France; Claude.Zoukimian@univ-grenoble-alpes.fr (C.Z.); remy.beroud@smartox-biotech.com (R.B.); 3 Department of Molecular Chemistry, Univ. Grenoble Alpes, CNRS, 570 rue de la chimie, CS 40700, 38000 Grenoble, France; didier.boturyn@univ-grenoble-alpes.fr; 4 Faculty of Medicine and Health Sciences, Department of Basic and Applied Medical Sciences, 9000 Gent, Belgium; frank.bosmans@ugent.be; 5 Toxicology and Pharmacology, University of Leuven, Campus Gasthuisberg, P.O. Box 922, Herestraat 49, 3000 Leuven, Belgium; eva.cuypers@kuleuven.be (E.C.); jan.tytgat@kuleuven.be (J.T.); 6 Université Côte d’Azur, CNRS UMR7275, Institut de Pharmacologie Moléculaire et Cellulaire, 660 route des lucioles, 6560 Valbonne, France; diochot@ipmc.cnrs.fr (S.D.); lazdunski@ipmc.cnrs.fr (M.L.); 7 Institute of Legal Medicine, University of Frankfurt, Kennedyallee 104, 60488 Frankfurt, Germany; mebs@em.uni-frankfurt.de; 8 Institute for Molecular Bioscience, University of Queensland, Brisbane 4072, Australia; d.craik@imb.uq.edu.au

**Keywords:** spider toxin, directed disulfide bond formation, Na_v_ channel activity, Na_v_1.7, pain target, automated patch-clamp

## Abstract

Phlotoxin-1 (PhlTx1) is a peptide previously identified in tarantula venom (*Phlogius* species) that belongs to the inhibitory cysteine-knot (ICK) toxin family. Like many ICK-based spider toxins, the synthesis of PhlTx1 appears particularly challenging, mostly for obtaining appropriate folding and concomitant suitable disulfide bridge formation. Herein, we describe a procedure for the chemical synthesis and the directed sequential disulfide bridge formation of PhlTx1 that allows for a straightforward production of this challenging peptide. We also performed extensive functional testing of PhlTx1 on 31 ion channel types and identified the voltage-gated sodium (Na_v_) channel Na_v_1.7 as the main target of this toxin. Moreover, we compared PhlTx1 activity to 10 other spider toxin activities on an automated patch-clamp system with Chinese Hamster Ovary (CHO) cells expressing human Na_v_1.7. Performing these analyses in reproducible conditions allowed for classification according to the potency of the best natural Na_v_1.7 peptide blockers. Finally, subsequent in vivo testing revealed that intrathecal injection of PhlTx1 reduces the response of mice to formalin in both the acute pain and inflammation phase without signs of neurotoxicity. PhlTx1 is thus an interesting toxin to investigate Na_v_1.7 involvement in cellular excitability and pain.

## 1. Introduction

Voltage-gated sodium (Na_v_) channels are critical for the generation and propagation of action potentials [1,2,3,4]. They are composed of a pore-forming α-subunit and can be associated with β-subunits [5]. Nine isoforms of vertebrate α-subunits have been identified so far (Na_v_1.1 to Na_v_1.9), that can be further distinguished by their sensitivity to tetrodotoxin (TTX); a toxin from the Japanese Puffer fish. Indeed, Na_v_1.5, 1.8 and 1.9 are TTX-resistant (TTX-r), whereas the other α-subunits are TTX-sensitive (TTX-s). These channels also differ by their distribution with Na_v_1.1, 1.2 and 1.3 principally found in the central nervous system, whereas Na_v_1.6, 1.7, 1.8 and 1.9 are predominantly, but not exclusively, expressed in the peripheral nervous system. In addition, Na_v_1.4 is predominantly found within the skeletal muscle, whereas Na_v_1.5 is chiefly present in cardiac muscle.

Na_v_ channels are involved in a wide array of physiological processes. In particular, Na_v_1.7 was clearly identified as playing a crucial role in nociceptive pathways, which led to research into the development of novel therapeutics for pain treatment [6,7,8,9,10,11,12,13,14,15,16,17,18]. For examples, missense mutations of the *SCN9A* gene that encodes Na_v_1.7 produces congenital indifference to pain [19]. Mice in which the *SCN9A* gene is inactivated produce a similar phenotype of pain resistance [20,21]. *A contrario*, gain of function mutations of *SCN9A* lead to the opposite spectra of clinical manifestations, including paroxysmal extreme pain disorder [22], painful small fiber neuropathy [23,24], iodiopathic small fiber neuropathy [2,25], or primary erythromelalgia with burning pain in extremities [26,27,28]. A set of biophysical alterations in Na_v_1.7 channel properties accompanies these pathologies, including changes in fast inactivation, the induction of persistent currents, and lower voltage thresholds for activation. Upregulation of Na_v_1.7 is also associated with metastatic potential in prostate cancer in vivo and could therefore be used as a putative functional diagnostic marker [29,30]. Finally, Na_v_1.7 may (i) have a role in the migration and cytokine responses of human dendritic cells [31]; (ii) help regulate neural excitability in vagal afferent nerves [32]; and (iii) contribute to odor perception in humans [33]. Despite its therapeutic potential, the task of identifying selective Na_v_1.7 channel inhibitors remain challenging given the high level of sequence homologies among Na_v_ channel isoforms, particularly in the structural loci governing ion conduction and selectivity. In spite of these difficulties, Xenome Pharmaceuticals successfully discovered XEN402, a compound that exhibits a voltage-dependent block of Na_v_1.7 and is capable of alleviating pain in erythromelalgia patients [34]. Researchers at Merck reported the discovery of a novel benzazepinone compound that blocks Na_v_1.7 and is orally effective in a rat model of neuropathic pain [35]. Finally, Genentech investigators found that aryl sulfonamide inhibitors selectively block Na_v_1.7 by a voltage-sensor trapping mechanism [36]. Due to their physiological importance, Na_v_ channels are also one of the foremost targets of animal venoms or plant neurotoxins [37,38,39]. The binding properties of peptide toxins have characteristic features that facilitate the identification of new Na_v_ channel isoform-selective pharmacological entities. For example, many of the peptidic toxins identified so far do not target the conserved pore region but rather act as gating modifiers by influencing movements of the less-conserved voltage-sensing domain within Na_v_ channels [40,41]. This feature further enhances the success rate for identifying Na_v_1.7 blockers with substantially improved selectivity over other Na_v_ isoforms. Reports on high affinity toxins for Na_v_1.7 that are efficient to treat pain remain infrequent. For instance, the tarantula venom peptide protoxin II (ProTx-II) potently inhibits Na_v_1.7 activation and abolishes C-fiber compound action potentials in desheathed cutaneous nerves [42]. However, ProTx-II application has little effect on action potential propagation of an intact nerve, an observation that may explain why ProTx-II is not efficacious in rodent models of acute and inflammatory pain. The scorpion toxin OD1 impairs Na_v_1.7 fast inactivation at low nanomolar concentrations but lacks the required Na_v_ channel isoform selectivity [43]. Finally, µ-SLPTX-Ssm6a from centipede venom was reported to block Na_v_1.7-mediated currents and produce favorable analgesic activity in a rodent model of chemical-induced, thermal, and acid-induced pain [44]; however, these data have yet to be reproduced. Part of the lack of efficacy of toxins on pain treatment can be explained by the fact that they require a concomitant activation of the opioid system to reveal their analgesic properties [45]. The theraphosidae family of spiders, belonging to the mygalomorph suborder, provided up to 20 analgesic peptides so far, all acting on Na_v_1.7, and belong to one of three spider toxin families (NaSpTx-1, NaSpTx-2 and NaSpTx-3) [46]. All these peptides vary in size from 26 to 35 amino acid residues, and are folded according to an inhibitor cysteine knot architecture with three disulfide bridges organized in a Cys^1^-Cys^4^, Cys^2^-Cys^5^, and Cys^3^-Cys^6^ pattern. As a rule, these peptides are difficult to fold and require expert chemical techniques for synthetic production. Altogether, because analgesic peptides are difficult to identify, it remains important to further enlarge the repertoire of Na_v_1.7-blocking toxins available to investigators interested in pain therapeutics.

Here, we report the chemical synthesis and the directed disulfide bridge formation of phlotoxin-1 (PhlTx1), a 34-residue and three disulfide-bridged toxin from the venom of a Papua New Guinea tarantula of a *Phlogiellus* genus spider species that was only scarcely characterized so far [47]. In addition, some synergistic effects were observed to occur between low doses of PhlTx1 and opioids for the treatment of inflammatory pain, suggesting an effect on Na_v_1.7 channel [45,48]. Herein, PhlTx1 was tested on a large array of ion channels including voltage-gated potassium (K_v_) channels, members of the two-pore domain potassium channel family (TASK1, TRAAK), inward-rectifying potassium channels, voltage-gated calcium (Ca_v_) channels and Na_v_ channels expressed in either Xenopus *laevis* oocytes, COS or CHO cells. Remarkably, the principal effect was seen on Na_v_ channels. In particular, Na_v_1.7 was found to represent the most sensitive isoform for PhlTx1 inhibition. We compared the Na_v_1.7 blocking efficacy of PhlTx1 to 10 other-published Na_v_1.7 blocking toxins that we chemically synthesized. Using an automated patch-clamp system with a single CHO expressing human Na_v_1.7 cell line, we ranked the potency of PhlTx1 activity on human Na_v_1.7 channel *versus* these other spider toxins in uniform experimental conditions. Finally, the analgesic potential of PhlTx1 was studied using the formalin pain test, indicating that it also represents an interesting lead compound for the development of an analgesic.

## 2. Results

### 2.1. PhlTx1 Description

**Figure 1:**
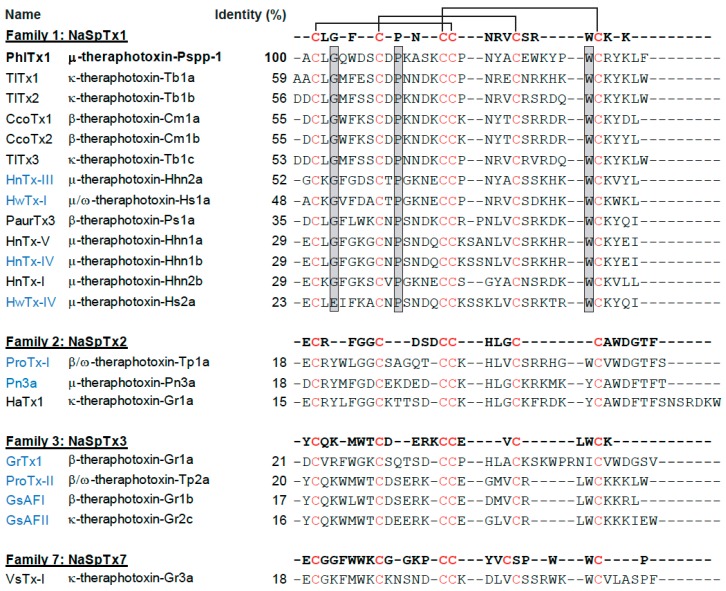
Sequence alignment of PhlTx1 with other spider toxins. Highly conserved cysteines are indicated in red and probable cysteine pairing, according to the inhibitory cysteine knot motif and the consensus sequence for NaSpTx-1 family of toxins, is indicated at the top with black lines. Percent conserved residues are indicated on the right. Shaded boxed residues correspond to the consensus residues of NaSpTx-1 toxin sequences. The lower homology with toxins from NaSpTx-2, NaSpTx-3 and NaSpTx-7 families are also shown. Toxins that are in blue have been compared in terms of activity on the Na_v_1.7 channel with PhlTx1 (see Figure 7).

PhlTx1 has been purified originally from the venom of *Phlogiellus* sp., *Theraphosidae Selenocosmiinae* (endemic to Papua New Guinea) and sequenced [47]. The peptide contains 34 amino acid residues, six cysteine residues bridged in an inhibitory cysteine-knot (ICK) architecture fold and is amidated at the C-terminus. The reported molecular weight of PhlTx1 is 4058.83 Da. Sequence alignment of PhlTx1 with other spider toxins illustrate that PhlTx1 has limited homology with previously identified peptides (sequence identities varying between 24 and 59% at best) (Figure 1). According to its sequence, PhlTx1 fits best within the NaSpTx-1 family with the following disulfide bridge organization: Cys^2^-Cys^17^, Cys^9^-Cys^22^, Cys^16^-Cys^29^. In contrast, it has very little homology with toxins from NaSpTx-2, NaSpTx-3 and NaSpTx-7 families (Figure 1). Several structural features of PhlTx1 hint at difficulties performing chemical synthesis. First, it contains three Pro residues (Pro^11^, Pro^18^ and Pro^27^) that are all susceptible to trans/cis isomerization and hence influence the proper induction of the secondary structures as well as the appropriate disulfide bridge pattern. Second, the immediate proximity of Cys^16^ and Cys^17^ is potentially a factor that could lead to disulfide bridge disarrangement, accompanied by inappropriate folding of the peptide and altered pharmacology. Finally, the lack of reporting on the chemical synthesis of PhlTx1, in spite of its potential interest (Na_v_1.7 target for pain treatment) and its discovery dating back to 2005 is a sign that its chemical synthesis is not straightforward.

### 2.2. PhlTx1 Chemical Synthesis

**Figure 2:**
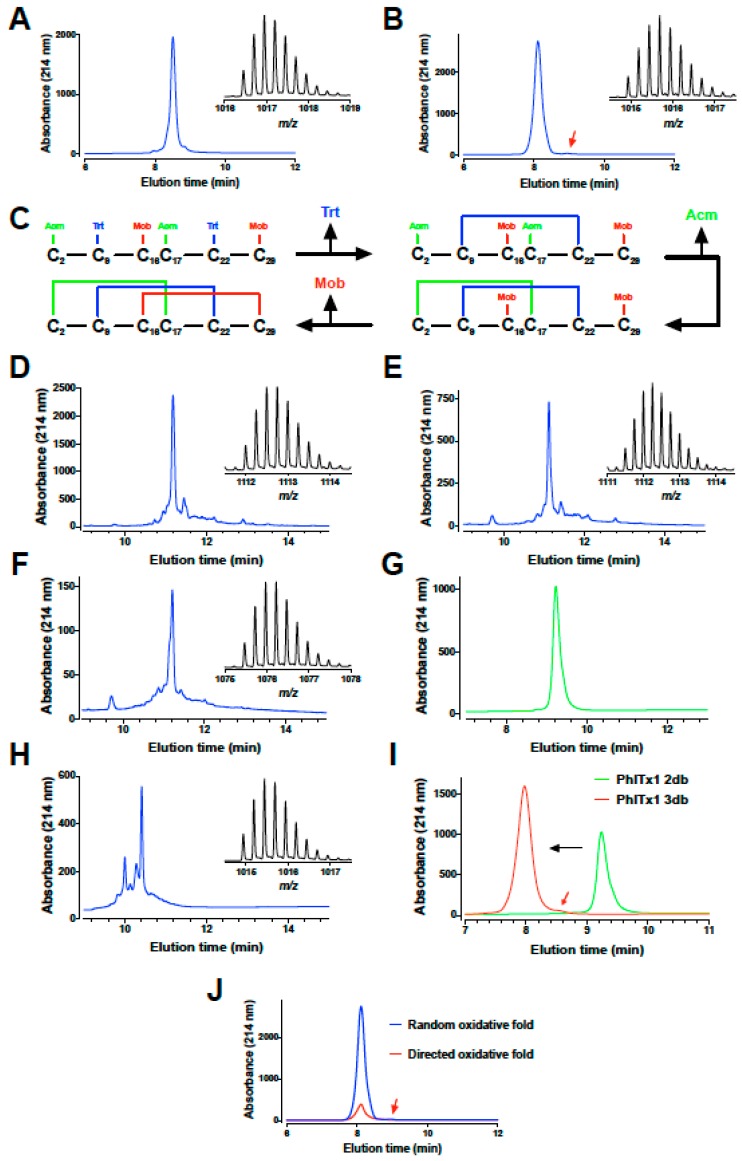
Comparative chemical synthesis of PhlTx1 following the random oxidative folding or the directed disulfide bond formation strategies. (**A**) Analytical RP-HPLC of the purified linear PhlTx1 meant to be produced by random oxidative folding (only Trt protecting groups for the 6 cysteine residues during chemical synthesis). Inset: MS spectrum of synthetic linear PhlTx1. [M + 4H]^4+^ of 1016.45. (**B**) Analytical RP-HPLC of the folded oxidized PhlTx1. Red arrow possibly indicates the second conformer of PhlTx1 that is present. Inset: MS spectrum of purified PhlTx1. [M+4H]^4+^ of 1014.94. (**C**) Schematic procedure employed for the directed disulfide bond formation strategy. (**D**) Analytical RP-HPLC of the crude linear PhlTx1 used for the directed disulfide bond formation strategy. Inset: MS spectrum of the synthetic compound with the Acm and Mob protecting groups. [M + 4H]^4+^ of 1111.99. (**E**) Analytical RP-HPLC of crude PhlTx1 with its first disulfide bridge. Inset: corresponding MS spectrum of the compound. [M + 4H]^4+^ of 1111.50. (**F**) Analytical RP-HPLC of crude PhlTx1 with its two first disulfide bridges. Inset: corresponding MS spectrum of the compound. [M + 4H]^4+^ of 1075.47. (**G**) Analytical RP-HPLC of purified PhlTx1 with its two first disulfide bridges. (**H**) Analytical RP-HPLC of crude PhlTx1 in its fully folded configuration. Inset: corresponding MS spectrum of the compound. [M + 4H]^4+^ of 1014.94. (**I**) (**G**) Analytical RP-HPLC of purified two-disulfide bridged (2db) and three-disulfide-bridged (3db) PhlTx1 to illustrate the important reduction of hydrophobicity of the peptide upon removal of the Mob protecting groups. (**J**) Coelution of purified PhlTx1 produced by random oxidative folding with that produced by a directed disulfide bond formation strategy.

In a first attempt to produce synthetic PhlTx1, the peptide was first stepwise assembled using fmoc chemistry, fully deprotected and purified from the crude synthetic products by preparative reversed-phase high pressure liquid chromatography (RP-HPLC) (Figure 2A). The purified linear peptide has the expected monoisotopic mass of 4061.79 (Figure 2A inset). Random oxidative folding of the peptide was performed at 0.1 mg/mL in a 100 mM Tris-HCl buffer at pH 8.4 with 5 mM reduced (GSH), 0.5 mM oxidized glutathione (GSSG) and 2 M Gn.HCl during 72 h at room temperature. According to the elution profile on RP-HPLC, the folding was (i) not straightforward, (ii) of very low yield and (iii) provided several peaks, possibly because of trans/cis isomerization properties of the Pro residues (data not shown). However, a dominant peak was purified as shown on the elution profile (Figure 2B). This purified peak resulted in the observation of two peaks in analytical RP-HPLC unless the column was heated, indicating the involvement of at least one Pro residue involved in trans/cis isomerization. Purifying either peak resulted in the production of the second peak demonstrating that the two forms (cis and trans) of the peptide are in equilibrium (data not shown). Other oxidative folded products may reflect misfolding of the peptide and inappropriate disulfide bridge arrangements. Purified folded/oxidized synthetic PhlTx1 was nevertheless shown to possess the proper monoisotopic mass of 4055.74 Da (Figure 2B inset). The 6 Da reduction in molecular weight of PhlTx1 is consistent with the formation of three disulfide bridges, but does not provide any indication about the favored pattern of disulfide bridges adopted by the toxin during this oxidative folding.

Because the random oxidative folding strategy and the final yield of production of PhlTx1 seemed problematic, we tried a directed disulfide bond formation strategy that ensures that the proper disulfide bridges are formed as expected for a NaSpTx-1 family toxin. Three different protecting groups were used for the lateral chains of Cys residues: Trt for Cys^9^-Cys^22^, Acm for both Cys^2^ and Cys^17^, and Mob for both Cys^16^ and Cys^29^. The sequential order of disulfide bridge formation was thus Cys^9^-Cys^22^ first after a classical deprotection of Trt groups with TFA, followed by Cys^2^-Cys^17^ second and lastly Cys^16^-Cys^29^ (Figure 2C). The two first disulfide bridges were sequentially formed in the same reaction buffer (one pot reaction), while the deprotection of Mob and the formation of the third disulfide bridge were done after purification of the two-disulfide-bridged PhlTx1. The synthetic crude PhlTx1 with the four remaining protecting groups (2 Acm and 2 Mob) after deprotection is shown on the RP-HPLC elution profile in Figure 2D, while the crude reaction mixture after formation of the first disulfide bridge is shown in Figure 2E. Formation of the first disulfide bridge is witnessed by the mass spectrometry analyses that illustrate a reduction in molecular weight (see insets; exact mass 4443.97 Da for the unfolded and 4441.96 Da for the one-disulfide bridged PhlTx1). The second disulfide bridge was formed after formation of the first one without intermediary purification in acidic conditions by the removal of the Acm protecting groups using iodine (see crude reaction mixture after formation of the second disulfide bridge in Figure 2F) and purified to homogeneity (Figure 2G). The acidic conditions were set to avoid disulfide bridge scrambling and secondary reactions with iodine such as iodine adducts on tyrosine or tryptophan residues. Next, the third disulfide bridge was formed in an oxidative and acidic buffer after removal of the Mob protecting groups with trifluoromethanesulfonic acid (TFMSA). Again, acidic conditions were used to avoid the risks of scrambling (see crude product in Figure 2H). The purified two-disulfide and three disulfide-bridged PhlTx1 are shown in Figure 2I. The large leftward shift observed in the retention time during elution is mainly due to the removal of the Mob protecting groups that are highly hydrophobic. All figure insets illustrating mass spectrometry profiles of the products indicate that each disulfide bridge has been formed properly with the appropriate removals of the protecting groups (exact mass of 4297.87 Da for the two disulfide-bridged PhlTx1 and 4055.73 Da for the fully-bridged disulfide PhlTx1). The overall yield of PhlTx1 production was 1.4%, which was 5 times better than that reached by random folding strategy. Nevertheless, the first strategy is expected to yield the same type of folding as both pure synthetic PhlTx1 coeluted perfectly (Figure 2J). This further illustrates that we purified the right peak within the random oxidative folding strategy, a fact that was not guaranteed in advance considering the presence of several peaks with the proper masses for oxidized PhlTx1. The directed disulfide bonding strategy we used should reveal itself to be very useful in experimental conditions where the random oxidative folding strategy turns to be inefficient (in SAR studies for example).

### 2.3. Ion Channel Selectivity of PhlTx1 and Preferential Activity on Na_v_1.7

**Figure 3:**
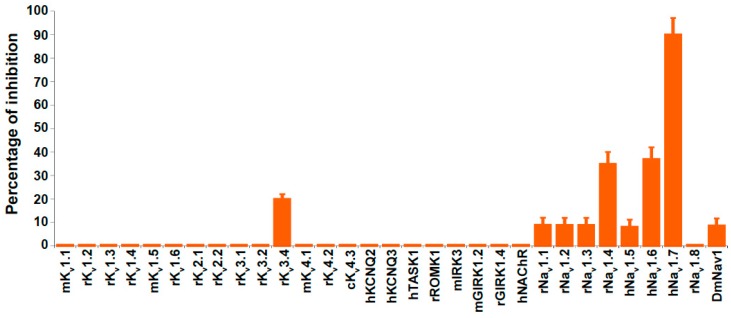
Overview of all tested channels indicating the percentage inhibition upon application of 1 µM PhlTx1. Expression system was either *Xenopus laevis* oocytes (all Na_v_ channels) and/or COS cells. Estimation of the effect on nicotinic acetylcholine receptor NAChR was achieved by ^125^I-α-bungarotoxin binding as previously described [49]. Clones were from the following species: m, mouse; r, rat; c, canine; h, human; and Para/tipE, Fruit fly.

We tested the biological activity of PhlTx1 on a large selection of ion channels that comprise K_v_ channels, two members of the two-pore domain potassium channel family (TASK1, TREK2), inward-rectifying potassium channels, the acetylcholine receptor and Na_v_ channels (Figure 3). All tested targets were heterologously expressed in either *Xenopus laevis* oocytes and/or COS cells and the resulting ionic currents were measured using electrophysiological voltage-clamp techniques. When applying 1 μM PhlTx1 to all tested ion channels and measuring current inhibition at the voltage of maximum ion flux, K_v_3.4 was the only non-Na_v_ channel impacted by the toxin (significant inhibition close to 20%). None of the channels tested were activated by the toxin.

**Figure 4:**
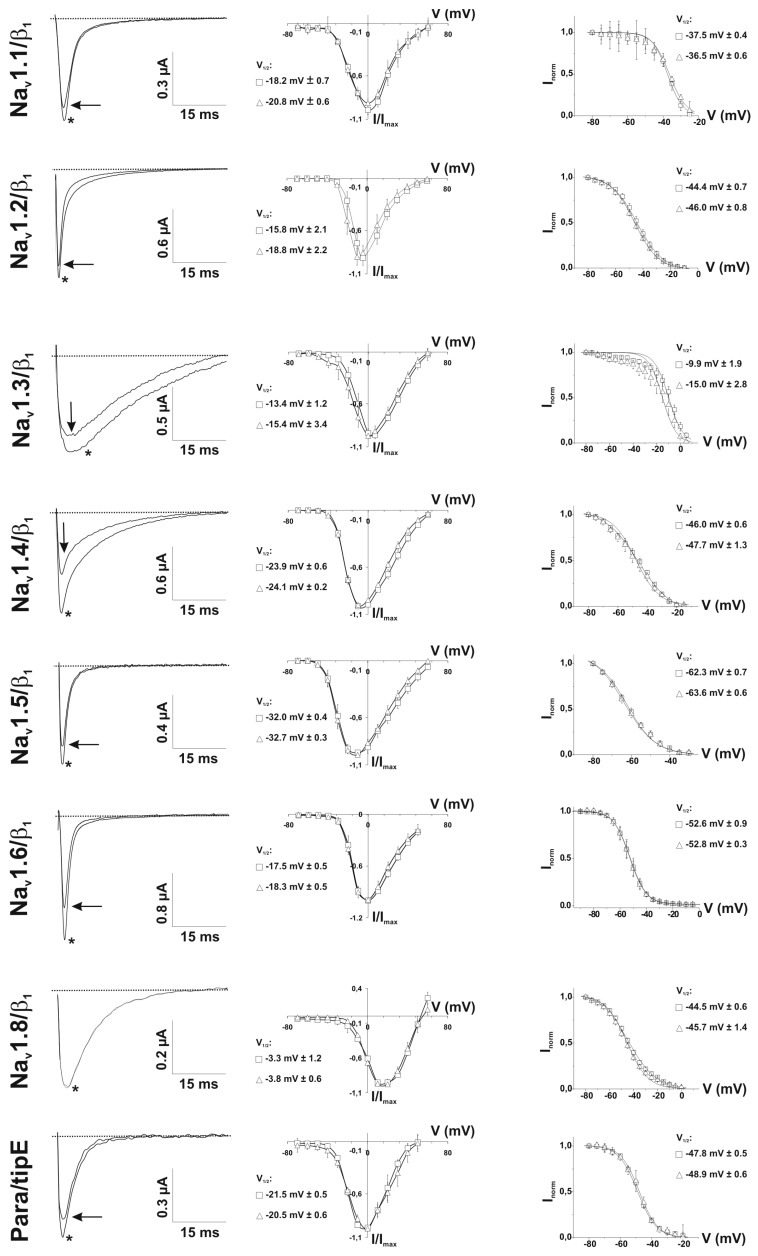
1 µM of PhlTx1 was tested on Na_v_1.1/β_1_, Na_v_1.2/β_1_, Na_v_1.3/β_1_, Na_v_1.4/β_1_, Na_v_1.5/β_1_, Na_v_1.6/β_1_, Na_v_1.8/β_1_ and para/tipE (*n* ≥ 3). Left column: current traces were evoked by a 50-ms depolarization to the voltage of maximum sodium influx, depending on the Na_v_ studied, from a holding potential of −90 mV. At 1 µM PhlTx1, all studied Na_v_ channels, except Na_v_1.4/β_1_, were blocked for about 10% (no toxin: *). The current of Na_v_1.4/β_1_ was reduced for about 35 ± 5%. Middle column: normalized I-V curves reveal no significant shift of the activation voltage (*p* > 0.05). Symbols: (□) before and (Δ) after addition of 1 µM PhlTx1. Right column: steady-state inactivation curves for all studied Na_v_ isoforms. No significant shift was observed for any of the channels (*p* < 0.05).

Using the two-electrode voltage-clamp technique on *Xenopus laevis* oocytes, the effect of PhlTx1 was also compared on eight different cloned vertebrate Na_v_ channels co-expressed with the β_1_ subunit (Na_v_1.1-1.8/β_1_) and on the neuronal insect Na_v_ channel, para, co-expressed with the tipE subunit (Figure 3 and Figure 4). When measured at the voltage of maximum sodium influx from a holding potential of −90 mV, 1 µM of PhlTx1 marginally decreased the sodium currents (10%) of most Na_v_ channel subtypes, while Na_v_1.8/β_1_ was not inhibited. Conversely, Na_v_1.4/β_1_ and Na_v_1.6/β_1_ currents revealed a maximum reduction of 35% when 1 µM PhlTx1 was applied (Figure 3). However, the inward currents of Na_v_1.7/β_1_ were almost completely inhibited (90 ± 7%) at 1 µM (Figure 3). Normalized current-voltage relationships (I-V curve) of Na_v_1.2/β_1_ and Na_v_1.3/β_1_ channels seemed to be shifted towards more negative potentials when PhlTx1 was applied, but shifts were not statistically significant (*p* < 0.05) (Figure 4). The activation phase of the other studied Na_v_ channel subtypes was not affected. No obvious alteration in channel availability was observed, except a 5-mV negative shift for Na_v_1.3/β_1_, although that was also non-significant (*p* > 0.05).

**Figure 5:**
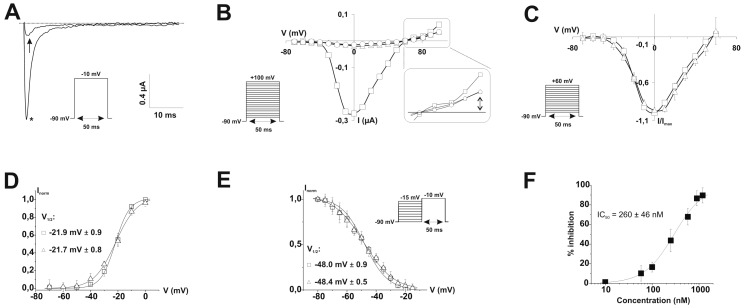
(**A**) Effect of 1 µM of PhlTx1 on Na_v_1.7/β_1_. Current trace was evoked by a 50-ms depolarization to −10 mV, from a holding potential of −90 mV. A nearly complete block of the inward sodium current is seen (no toxin: *). (**B**) I–V protocol to positive voltages (+100 mV). □ represents control conditions where no toxin was added. 50 nM of TTX was added (○) after maximum block was obtained with 1 µM of PhlTx1 (Δ). No further reduction of the outward current was seen (see inset). Therefore, the remaining outward current does not contain a Na_v_1.7/β_1_ component anymore. (**C**) Normalized I-V curve of Na_v_1.7/β_1_ before (□) and after addition of 1 µM PhlTx1 (Δ) (*n* = 5). (**D**) Normalized activation curves derived from (**C**). No change in activation voltages or V_1/2_ was seen. (**E**) Steady-state inactivation curves before and after addition of 1 µM PhlTx1. No effect was seen. (**F**) In order to obtain the IC_50_ value of PhlTx1 on Na_v_1.7/β_1_, the percentage of toxin-induced block was plotted against the concentration of toxin used and a fit with the Hill equation yielded a value of 260 ± 46 nM (*n* = 4; Hill coefficient = 1.3). The stimulus frequency was 0.3 Hz.

We further characterized the inhibition of Na_v_1.7/β_1_ currents by PhlTx1. The residual inward current after at 1 µM PhlTx1 addition seems to occur without any alteration in channel kinetics (Figure 5A). Outward sodium currents at depolarizing voltages (+100 mV) were also blocked, as assayed with the help of tetrodotoxin (TTX; 50 nM) which physically occludes the pore (Figure 5B). Furthermore, Na_v_1.7/β_1_ inhibition did not seem to be very voltage-dependent, since the reduction of sodium currents at more hyperpolarized voltages (−70 to −30 mV) was similar to the reduction of sodium current at more depolarized potentials (−20 to 60 mV). The Na_v_1.7/β_1_ gating parameters, before and after addition of 1 µM PhlTx1 (*n* = 4; Figure 5C), are not affected (*p* < 0.05; see Figure 5D). Again, no alteration in steady-state inactivation or E_rev_ was seen (Figure 5B,E). In order to obtain the IC_50_ value of PhlTx1 on Na_v_1.7/β_1_, expressed in *Xenopus laevis* oocytes, the percentage of toxin-induced block obtained at a stimulus frequency of 0.3 Hz was plotted against the concentration of toxin used and a fit with the Hill equation yielded a value of 260 ± 46 nM with a Hill coefficient = 1.3 (Figure 5F).

### 2.4. Refined Affinity of PhlTx1 for the Na_v_1.7 Channel in Mammalian Cells

**Figure 6:**
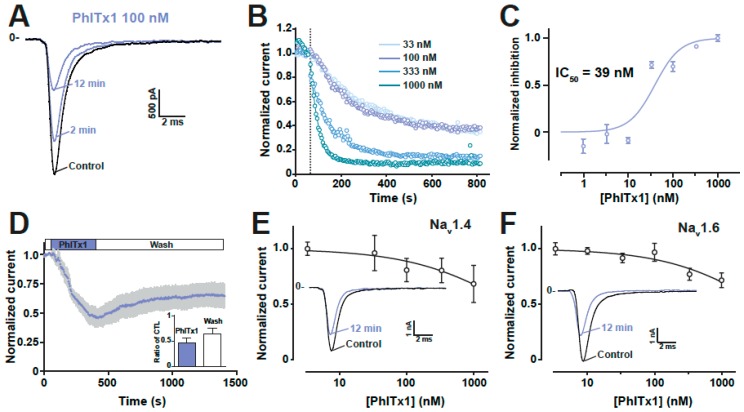
PhlTx1 inhibition of Na_v_1.7 channels expressed in a mammalian cell line. (**A**) Representative current traces of Na_v_1.7 current elicited at -10 mV (holding potential = −100 mV) before (Control) and at various times after application of 100 nM PhlTx1 (2 min and 12 min). (**B**) Average normalized current amplitudes before (left from vertical dotted line) and after application of various concentrations of PhlTx1 (right of dotted line). The average data points were fitted by mono-exponential decay equation y = 1-exp-t/τ + c with τ being the time. A c value of 0.09 at 1 μM PhlTx1 indicates that the block induced by PhlTx1 is not complete. A plateau of inhibition was reached for all concentrations. (**C**) Average dose-response curve of hNa_v_1.7 current block by PhlTx1. A sigmoid Hill fit provides an IC_50_ value of 39 ± 2 nM and a Hill value of 1.53. Numbers of cells per concentration: 4 to 8. (**D**) Average kinetics of reversibility of PhlTx1 block of hNa_v_1.7 currents (*n* = 5 cells). (**E**) Average dose-response curve of hNav1.4 block by PhlTx1. Numbers of cells per concentration: 4 to 13. (**F**) Average dose-response curve of hNav1.4 block by PhlTx1. Numbers of cells per concentration: 15 to 23.

Following our initial screen for the selectivity of synthetic PhlTx1 and the discovery that Na_v_1.7 was the main target, we decided to further evaluate the activity of PhlTx1. We used a CHO mammalian cell line expressing the human Na_v_1.7 instead of *Xenopus* oocytes for the pharmacological evaluation. To this end, we used an automated patch-clamp system, the Nanion syncropatch 384PE, and a standardized robotic method from Beckmann to apply PhlTx1 in the recording chambers and collect the kinetic information of Na_v_1.7 current from a high number of Na_v_1.7-expressing cells. The syncropatch 384PE has the potential to record from 384 cells at a time. 100-ms pulses from −100 mV to −10 mV were applied at a frequency of 0.2 Hz and various concentrations of PhlTx1 were applied to the cells. An example of a Na_v_1.7 current recording is illustrated in Figure 6A along with the effect of 100 nM PhlTx1. It is of interest to note that current inhibition develops over time at this PhlTx1 concentration that is above 50% at the end of a 13-min application time. As in oocytes experiments, PhlTx1 does not affect the kinetics of Na_v_1.7 currents (activation or inactivation). We illustrated the time course of current block by PhlTx1 at concentrations (33 nM, 100 nM, 333 nM and 1 μM) that best frame the inhibition of Na_v_1.7-mediated currents (Figure 6B). Note that the highest achievable inhibition was 85%–92% for the two most efficient concentrations (333 nM and 1 μM). The time constant of inhibition was concentration-dependent as expected and could be fitted with decreasing mono-exponentials with time constants of 253 s (33 nM, *n* = 4), 159 s (100 nM, *n* = 8), 97 s (333 nM, *n* = 6) and 36 s (1 μM, *n* = 8). Reporting the maximally reached inhibition at the end of the PhlTx1 application as a function of PhlTx1 concentration indicates that PhlTx1 inhibits Na_v_1.7 currents with an IC_50_ value of 39 ± 2 nM (Figure 6C). Using a manual patch clamp, which is better suited to investigate current recovery from block than an automated patch clamp that requires several washing steps, we investigated the reversibility properties of a PhlTx1 block. As shown, the toxin effect was partially and poorly reversible (Figure 6D). After 16 min of washout of 100 nM PhlTx1, we observed a maximum of 32% recovery of the blocked current that occurred with a time constant of 303 s. Since Na_v_1.4 and Na_v_1.6 were the two most sensitive channels to the PhlTx1 block, we also performed dose-response curves for these two channels with the automated patch clamp system. According to the fits of the data, the calculated IC_50_ values of PhlTx1 for hNa_v_1.4 and hNa_v_1.6 expressed in CHO and HEK293 cells, respectively, were above 3 μM, indicating that PhlTx1 was indeed quite selective for the human Na_v_1.7 channel (Figure 6E,F).

### 2.5. Comparison of the Na_v_1.7 Channel Blocking Efficience of PhlTx1 with That of Leading Toxins Active on Na_v_1.7

**Figure 7:**
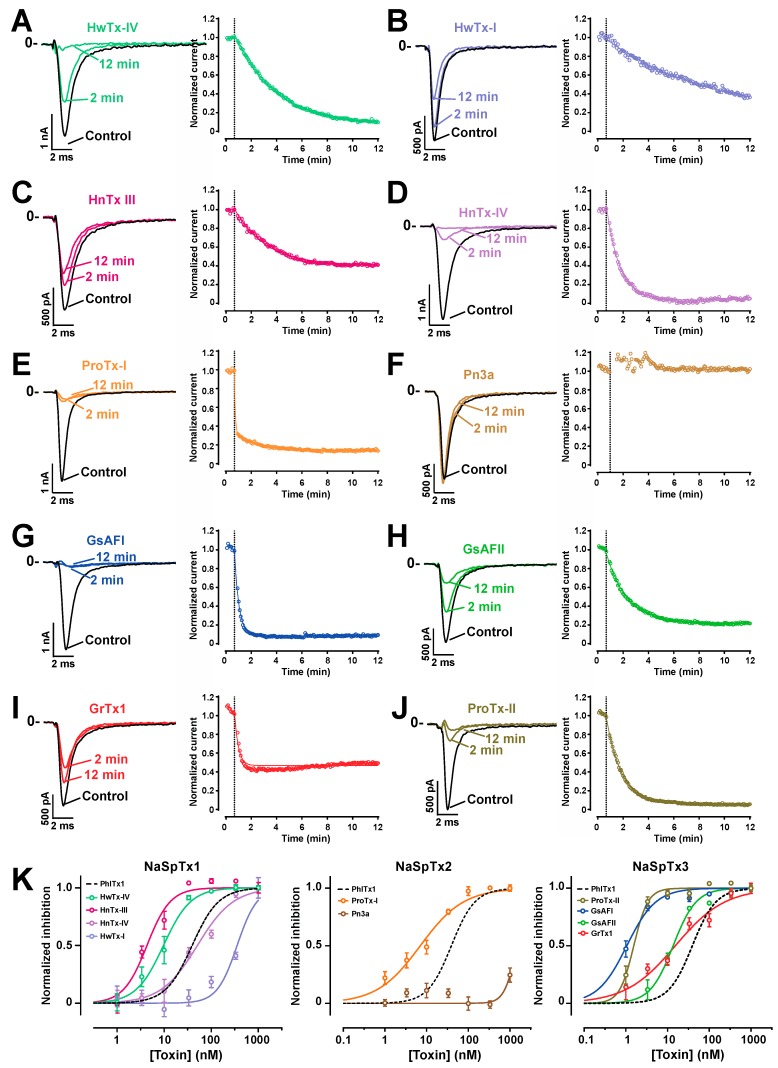
Comparison of PhlTx1-mediated inhibition of Na_v_1.7 currents with the inhibition mediated by other reported Na_v_1.7-blocking toxins. (**A**–**J**) Ten synthetic toxins, reportedly active on Na_v_1.7 channel, from three families (NaSpTx1, NaSPTx2 and NaSpTx3) were tested for their blocking potency at 100 nM. Representative traces elicited from a holding potential of -100 mV and test pulse −10 mV in control condition and 2 and 12 min of toxin application time (left panels). All toxins were active at 100 nM with the exception of Pn3a. The kinetic of Na_v_1.7 current block are shown in the right panels, thereby differentiating the fast and slow blocking toxins at this 100-nM concentration. (**K**) Normalized dose-response curves of current inhibition of each toxin compared to PhlTx1 for each NaSpTx family. Inhibitions were measured at end of a 12- or 15-min application time depending on toxin properties (fast or slow blocking). IC_50_ and Hill values obtained were 7.1 ± 1.2 nM and 0.84 (ProTx-I; *n* = 4–8 cells per point), 1.0 ± 1.1 nM and 1.2 (GsAFI; *n* = 5–8 cells per point), 13.6 ± 1.2 nM and 1.6 (GsAFII; *n* = 2–8 cells per point), 1.5 ± 1.1 nM and 2.5 (ProTx-II; *n* = 5–8 cells per point), 4.3 ± 1.1 nM and 1.74 (HnTx-IV; *n* = 5–8 cells per point), 15.3 ± 1.2 nM and 0.73 (GrTx1; *n* = 5–8 cells per point), 1457 ± 169 nM and 3 (Pn3a; *n* = 3–7 cells per point), 50.6 ± 1.2 nM and 1.1 (HnTx-III; *n* = 5–8 cells per point), 25.1 ± 1.1 nM and 2.3 (HwTx-I; *n* = 2–7 cells per point), and 9.6 ± 1.2 nM and 1.5 (HwTx-IV; *n* = 6–8 cells per point). IC_50_ values of HwTx-IV, HwTx-I and HnTx-III are likely to be slightly under-evaluated because of incompletely reaching equilibrium at the lowest effective concentrations. The block by PhlTx1 is shown for comparison (dashed line).

PhlTx1 has two important properties underpinning its potential to be developed as analgesic: (i) a suitable selectivity profile, and (ii) a high affinity for Na_v_1.7. Therefore, it was of interest to test how PhlTx1 ranks in terms of Na_v_1.7 blocking activity compared to some leading peptides that are published as being active on this channel type [50,51,52,53]. Comparing data from published information is challenging as often the experiments are performed in different experimental conditions (including different cell lines, different species from which the Na_v_1.7 clone originates, as well as differing application conditions and chemical synthesis quality, or even if the toxin is of natural or synthetic origin). Thus, we decided to chemically synthesize 10 additional toxins that are reported to block Na_v_1.7 from various NaSpTx families (1, 2 and 3) and to test them in similar experimental conditions on the CHO cell line expressing Na_v_1.7. Representative Na_v_1.7-mediated currents traces are shown for each toxin type along with the extent of current block by 100 nM of this toxin at two time points (left panels of Figure 7A–J). Several observations can be made. ProTx-I, GsAFI, ProTx-II and HnTx-IV all lead to both relatively rapid and almost a complete block of Na_v_1.7 current. The average time courses of inhibition are shown on the right panels for a concentration of 100 nM; a concentration set arbitrary that we consider as a good cut-off concentration to decide whether a toxin is an interesting lead blocker for Na_v_1.7. At this concentration, all toxins block over 50% of the inward current with the exception of Pn3a; this toxin being fully insensitive at this concentration. The block by GrTx1 reached a plateau of inhibition of about 50% on average. The toxins differed also by their time course of block. At 100 nM, the fastest blocking toxins were ProTx-I (double exponential time course of inhibition with time constants of τ_1_ = 4 s and τ_2_ = 114 s), GsAF1 (τ = 21 s) and GrTx1 (τ = 18 s). Slower, but complete blocks, were observed for HwTx-IV (τ = 194 s), HnTx-III (τ = 149 s), HnTx-IV (τ = 62 s), GsAFII (τ = 98 s) and ProTx-II (τ = 72 s). The slowest blocking toxin was HwTx-I with an incomplete block even after 11 min 20 sec of application (τ = 492 s). The block by GrTx1 was accompanied by a slight current recovery at -10 mV test potential after it reached its maximal block. This is most likely due to a secondary alteration of the voltage-dependence of activation by the toxin, possibly by binding on a second site of lower affinity. This finding is in agreement with the concept of multimodal action of Tarentula toxins developed earlier, including for GrTx1 [54]. Finally, to fully characterize these peptides, we reported the blocking efficacies as a function of toxin concentrations (Figure 7K). The dose-response curves were illustrated for each NaSpTx families (NaSpTx-1: left panel; NaSpTx-2: middle panel; and NaSpTx-3: right panel). As illustrated, within the first family of toxins, PhlTx1 inhibition was most comparable to the inhibition by HnTx-III or HwTx-I (IC_50_ of 39 nM compared to 50.6 nM for HnTx-III or 25.1 nM for HwTx-I). Two toxins outperformed PhlTx1: HnTx-IV with an IC_50_ of 4.3 nM and HwTx-IV with an IC_50_ of 9.6 nM. Within the NaSpTx2 family, ProTx-I was 5.5-fold better than PhlTx1 with an IC_50_ of 7.1 nM, but Pn3a, reportedly very efficient, surprisingly had an estimated IC_50_ of 1457 nM. Within the NaSpTx3 family, all toxins were between 2.6- and 39-fold more active than PhlTx1 (IC_50_ values between 1 nM for GsAFI and 15 nM for GrTx1). The inhibition efficiency of GsAFI was unexpectedly even better than that of ProTx-II, the reference toxin in the literature for blocking Na_v_1.7 channels. Several of these findings are in contrast with published reports demonstrating the importance of a standardized procedure to properly compare the potency of all these toxins (Table 1).

With the exception of ProTx-II, none of the IC_50_ values we found matched those of the literature. We systematically found better values by an order of 2.7- (HwTx-IV) to 76-fold (GsAFI). The differences in affinity observed for GsAFI, GsAFII and GrTx1 are intriguing. In our analyses, these compounds were 40-, 76- and 25-fold more active in our hands than in an earlier report [54]. Here, two factors may explain the observed differences: (i) lack of use of BSA to avoid non-specific sticking of the peptides to the plastic tubes and dishes, and (ii) the fact that purified peptides were used and not synthetic ones. Quantifying native peptides is more difficult than quantifying synthetic ones because of limited quantities of material and is frequently a source of mistake in defining IC_50_ values. The 25-fold difference in IC_50_ value for HwTx-I also demonstrates the importance of standardized protocols for affinity measurements. *A contrario*, the most surprising finding was that Pn3a is far less active than expected [45]. The reason for this discrepancy is unclear, particularly since the chemical synthesis was straightforward. A more in-depth analysis of the disulfide bridge pattern acquired during the synthesis, possibly at odds with what was obtained earlier, may come as an explanation at a later stage.

### 2.6. Analgesic Potential of PhlTx1 In Vivo

**Figure 8:**
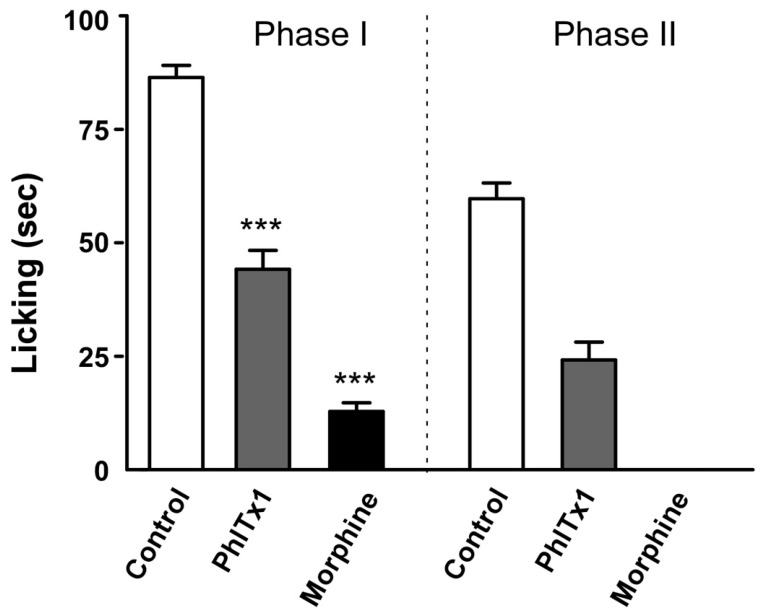
PhlTx1 is effective in a mouse model of acute and inflammatory pain. Effects of PhlTx1 (100 pmoles) and morphine (0.25 mg) on first (0–10 min) and second (10–45 min) phase of formalin-induced spontaneous pain behavior (*n* = 6–11) in mice. The licking value for morphine is 0 during phase II for all mice. Comparisons are *versus* vehicle unless specified. Mean ± s.e.m. *** = *p* < 0.001. This figure was presented in an earlier meeting in 2006 [47].

Given the role of Na_v_1.7 in nociception, we next evaluated the analgesic activity of PhlTx1 in an inflammatory pain assay on mice and compared the results to those obtained with morphine. Mice were injected intrathecally with vehicle solution only (0.9% NaCl, *n* = 11), morphine (0.25 mg in 10 µL vehicle, *n* = 6) or toxin-containing solution (100 pmoles PhlTx1 in 10 µL vehicle, 0.47 µg/mouse, *n* = 5). Rating of formalin-induced behavior was performed according to the time spent lifting, licking or biting the affected paw. In all cases, no neurotoxicity was observed after intrathecal injection. Strikingly, application of PhlTx1 substantially reduced the response of mice in both the acute pain (Phase I) and inflammation (Phase II) phases induced upon formalin injection into the paw, thereby demonstrating the analgesic effect of the toxin, possibly via a mechanism involving Na_v_1.7 inhibition (Figure 8). This is the first formal description of the anti-nociceptive potential of PhlTx1. Although it appears to contradict a previous report suggesting a lack of effect of PhlTx1 on acute pain [45], it must be emphasized that the routes of administration differed (intrathecal here *versus* intraperitoneal in the previous study).

## 3. Discussion

In this report, we illustrated that PhlTx1 is a new member of the NaSpTx-1 family of toxins active on Na_v_ channels. Since its discovery in 2005, reports on the use of PhlTx1 have been scarce [45,47] and an extensive description of its pharmacology has been lacking. Part of the reason for this lack of published information, at a time where the Na_v_1.7 has become a very interesting target for pain treatment, may lie in the difficulties linked to its chemical synthesis. Although the random oxidative folding strategy seems to work, the yields remain largely unsatisfactory to envision any kind of clinical future for this peptide or even to perform a complete Ala scan of the peptide to identify the pharmacophore of PhlTx1. Taking for granted that PhlTx1 folds according to the disulfide bridge motif of the NaSpTx-1 family, we decided to produce the toxin using a directed disulfide bond formation strategy. This strategy ensured that the disulfide bond pattern was respected and unique, led to a better yield of peptide production, and enforced the proper production of toxin variants that otherwise would fail to fold or oxidize properly. Concerning the wild-type sequence of PhlTx1, we do believe however that the random oxidative folding does yield the proper fold and disulfide bridges as both synthetic products coelute.

Although many new toxins are discovered each year, the same questioning regarding the natural target and the potential applications arises systematically. In many cases it is not sufficient to perform a blast search and find some homologous peptides, as this may be insufficient information to determine the target of the toxin. Here, PhlTx1 sequence is quite novel as its sequence barely matches other spider toxins. The fact that PhlTx1 belongs to the NaSpTx-1 family is also not sufficient information to indicate its potential cellular target. For this reason, we performed extensive testing of PhlTx1 activity on several voltage-dependent and one ligand-gated ion channels. Remarkably, we identified Na_v_1.7 as the most sensitive ion channel for PhlTx1, with very few additional ion channels being sensitive to this toxin. Two other Na_v_ channels, Na_v_1.4 and Na_v_1.6, showed interesting sensitivities as well to PhlTx1, that were better evidenced using mammalian cells than oocytes. The discrepancy in IC_50_ value for PhlTx1 blockage observed between oocytes and mammalian cells is a quite common phenomenon. It finds its origin in multiple factors such as lipid composition, the presence or absence of auxiliary subunits, or the fact that oocytes possess an extra vitelline membrane that peptides have to cross to reach their target. The rather narrow selectivity profile places PhlTx1 as an interesting lead candidate for the development of an analgesic. A careful reexamination of its affinity for the human Na_v_1.7 channel isoform, expressed in mammalian cells, further reveals that its affinity is below 100-nM. This issue is particularly important if this toxin needs to be further investigated for its analgesic properties considering that its chemical synthesis is not straightforward. High affinity toxins may benefit from the fact that lower amounts need to be used in vivo to become efficient in their therapeutic task. 

The modus operandi of channel block by PhlTx1 leads to some thoughts on its potential binding site. At face value, PhlTx1 activity resembles that of TTX that inhibits the pore. Other spider toxins such as HnTx-I, HnTx-III, HnTx-IV, HnTx-V and HwTx-IV also reduce the current amplitude of TTX-sensitive Na_v_ channels and have therefore been hypothesized to occlude the ion conduction pathway. HnTx-III, HnTx-IV and HnTx-V shift the voltage midpoint of steady-state inactivation to more hyperpolarized potentials, an effect that is not described with TTX when tested in rabbit purkinje fibers or here with PhlTx1. However, it is worth noting that most gating-modifier toxins interact with one or more voltage-sensing domains to inhibit or activate the channel [58]. Therefore, future work on PhlTx1 will include the determination of its binding site.

## 4. Conclusions

In summary, we successfully synthesized substantial quantities of the tarantula venom toxin PhlTx1 using two unrelated procedures that should greatly facilitate future SAR studies. The synthetic PhlTx1 is biologically active and was shown to predominantly inhibit Na_v_1.7-mediated currents in two heterologous expression systems, whereas a large collection of other ion channels is not or only slightly influenced. When tested in a mouse model for inflammatory pain, PhlTx1 reduced the response of mice in the acute pain and inflammation phases, thereby supporting the role of Na_v_1.7 in pain perception. Although PhlTx1 is not the most potent Na_v_1.7 ligand isolated to date, it is not a weak affinity blocker according to our comparative analyses with leading Na_v_1.7 acting toxins. In addition, it can reasonably be assumed that its affinity towards the channel may be improved further. For example, accumulating evidence suggests that defined peptide modifications can result in drastically increased toxin potencies [59,60,61]. The observation that Na_v_1.7 loss-of-function in humans does not induce mortality offers tantalizing prospects for finding new routes of analgesia [19]. PhlTx1, as a selective antagonist of Na_v_1.7, should therefore be considered as another exciting lead for novel pain treatments or as a potent pharmacological tool to dissect Na_v_1.7 contribution to cellular excitability.

## 5. Materials and Methods 

### 5.1. Chemicals and Peptides

The following toxins: ProTx-I, ProTx-II, GsAFI, GsAFII, GrTx1, Pn3a, HnTx-III, HnTx-IV, HwTx-I and HwTx-IV were all chemically assembled and provided by Smartox Biotechnology (Saint-Egrève, France).

### 5.2. Chemical Synthesis of PhlTx1

Linear PhlTx1 was assembled stepwise using fmoc solid-phase chemistry on a Symphony Synthesizer (Protein Technologies Inc., Tucson, AZ, USA) at a 0.1 mmol scale on 2-chlorotrityl chloride resin (substitution approx. 1.6 mmol/g). Fmoc protecting groups were removed using 20% piperidine in dimetilformamid (DMF) and free amine was coupled using tenfold excess of Fmoc amino acids and HCTU/DIEA activation in NMP/DMF (3 times 15 min). For the random oxidative folding strategy, all cysteine residues were introduced with trityl protecting groups. For the directed disulfide bond formation strategy, Cys^2^ and Cys^17^ were introduced with Acm protecting groups, Cys^9^ and Cys^22^ with trityl protecting groups, and Cys^16^ and Cys^29^ with Mob protecting groups. Linear peptides were deprotected and cleaved from the resin with TFA/H_2_O/1,3-dimethoxybenzene/TIS 92.5/2.5/2.5/2.5 (vol.), then precipitated out in cold diethyl ether and the resulting white solids were washed twice with diethyl ether to afford crude linear peptides. Next, for random oxidative folding strategy, the fully deprotected PhlTx1 was purified by preparative reversed-phase (RP) HPLC prior to oxidative folding. Purification by RP-HPLC on a C18 (10 µm, 100 Å) Phenomenex Luna stationary phase on an Agilent Technologies preparative HPLC system (eluent system H_2_O/MeCN + 0.1% TFA), afforded pure linear PhlTx1, which was folded by air oxidation at 0.1 mg/mL in a 100 mM Tris buffer at pH 8.4, containing 5 mM GSH, 0.5 mM GSSG and 2 M Gn.HCl. After 72 hrs at room temperature, the pH of the reaction mixture was adjusted to 3.0 and purified by preparative RP-HPLC. Two purifications, firstly by RP-HPLC on a C18 (10 µm, 100 Å) Phenomenex Luna stationary phase on an Agilent Technologies preparative HPLC system (eluent system H_2_O/MeCN + 0.1% TFA), then secondly on a C12 (4 µm, 90 Å) Phenomenex Proteo Jupiter stationary phase on a semi-preparative HPLC system (eluent system H_2_O/MeCN + 0.1% TFA), afforded pure synthetic PhlTx1 in a 0.25% overall yield. For the directed disulfide bond formation strategy, crude PhlTx1 was dissolved in H_2_O/MeCN (1:1) at 10 mg/mL and added dropwise to a solution containing 0.1 M citric acid and 10% DMSO, at pH 7.8, to a final peptide concentration of 0.1 mg/mL. After one night under gentle stirring, pH was adjusted to 1–2, and 1 eq. of 50 mM iodine in MeCN was added every five minutes, for a total of five additions. Five minutes after the last addition, the excess of iodine was quenched with sodium ascorbate and the solution was filtered and purified by preparative RP-HPLC. Purification by RP-HPLC on a C18 (10 µm, 100 Å) Phenomenex Luna stationary phase on an Agilent Technologies preparative HPLC system (eluent system H_2_O/MeCN + 0.1% TFA) afforded the pure two-disulfide bond PhlTx1. The freeze-dried peptide was dissolved in TFA/phenol at 0 °C and TFMSA was added to reach a concentration of 5 mg/mL of peptide in TFA/phenol/TFMSA (8:1:1). The mixture was stirred for 10 min at 0 °C and then the peptide was precipitated out in cold diethyl ether and the resulting white solid was washed twice with diethyl ether. The peptide was dissolved in H_2_O/MeCN (1:1) at 10 mg/mL and added dropwise to a solution containing 0.1 M citric acid, 15% DMSO and 2 M Gn.HCl at pH 2.0, to a final peptide concentration of 0.1 mg/mL. After 48 h, the solution was purified by RP-HPLC on a C18 (4 µm, 90 Å) Phenomenex Proteo Jupiter stationary phase on an Agilent Technologies preparative HPLC system (eluent system H_2_O/MeCN + 0.1% TFA) to afford pure synthetic PhlTx1 in a 1.4% overall yield.

### 5.3. Cell Culture

CHO cells stably expressing the human Na_v_1.7 cells were cultured in Dulbecco’s Modified Eagle’s Medium (DMEM) supplemented with 10% fetal calf serum, 1 mM pyruvic acid, 4 mM glutamine, 10 IU/mL penicillin and 10 μg/mL streptomycin (Gibco, Grand Island, NY, USA), and incubated at 37 °C in a 5% CO_2_ atmosphere. For automated patch-clamp recordings, cells were detached with trypsin and floating single cells were diluted (~300,000 cells/mL) in medium contained (in mM): 4 KCl, 140 NaCl, 5 Glucose, 10 HEPES (pH 7.4, osmolarity 290 mOsm).

### 5.4. Xenopus Oocyte Expression and Recording Experiments

For in vitro transcription, Na_v_1.5/pSP64T and Na_v_1.8/pSP64T were first linearized with *Xba*I and β_1_/pSP64T with *Eco*RI. Capped cRNAs were synthesized from the linearized plasmid using the SP6 mMESSAGE-mMACHINE^®^ transcription kit (Ambion, USA). The Na_v_1.1/pLCT1, Na_v_1.2/pLCT1, Na_v_1.3/pNa3T, Na_v_1.4/pUI-2, para/pG19-13-5 and tipE/pGH19 vector were linearized with *Not*I and Na_v_1.7/pBSTA.rPN1 was linearized with *Sac*II. Transcriptions were performed with the T7 mMESSAGE-mMACHINE^®^ kit (ThermoFisher Scientific, Illkirch, France). The harvesting of stage V-VI oocytes from the ovarian lobes of anaesthetized female *Xenopus laevis* frogs was carried out as previously described [62,63]. The use of *Xenopus laevis* was approved by the Animal Care Committee of the University of Leuven. Oocytes were injected with 50 nL of cRNA at a concentration of 1 ng nL^−1^ using a Drummond (USA) micro-injector. The ND96 solution used for incubating the oocytes contained (in mM): NaCl 96, KCl 2, CaCl_2_ 1.8, MgCl_2_ 2 and HEPES 5 (pH 7.4), supplemented with 50 mg L^−1^ gentamycin sulfate and 180 mg L^−1^ theophyllin. Two-electrode voltage-clamp (TEVC) recordings were performed at room temperature (19–23 °C) using a GeneClamp 500 amplifier (Molecular Devices, USA) controlled by a pClamp data acquisition system (Molecular Devices, USA). Whole-cell currents from oocytes were recorded 2 to 4 days after injection. Voltage and current electrodes were filled with 3 M KCl. Resistances of both electrodes were kept as low as possible (<0.5 MΩ). Bath solution composition was (in mM): NaCl 96, KCl 2, CaCl_2_ 1.8, MgCl_2_ 2 and HEPES 5 (pH 7.4). Currents were filtered at 1 kHz with a four-pole low-pass Bessel filter, and sampled at 5 kHz. PhlTx1 was dissolved in ND96 containing 0.1% bovine serum albumin (BSA). This stock solution was added to the bath solution at the concentrations indicated. Data manipulation was performed in pClamp8 (Molecular Devices, USA) and Origin software (MVB Scientific, Nes-Ameland, The Netherlands). Averaged data are presented as mean ± SEM. In general, current-voltage relationships (I-V curves) were evoked in oocytes expressing the cloned Na_v_ channels by 50-ms depolarizations between −70 to +120 mV, using 5 or 10 mV increments from a holding potential of −90 mV. To avoid overestimation of a potential toxin-induced shift in the current-voltage relationship due to inadequate voltage control when measuring large sodium currents in oocytes, only results from cells with currents lower than 1.5 µA were considered. In order to obtain IC_50_ values, the percentage of toxin-induced block was plotted against the concentration of toxin used. A fit with the Hill equation yielded the IC_50_ values.

### 5.5. Pharmacological Applications Using the Automated Patch-Clamp System

PhlTx1 and other Na_v_1.7-acting toxins were investigated on CHO cells expressing the human Na_v_1.7 channel using the Automated patch-clamp system from Nanion (München, Germany), the SyncroPatch 384PE. Chips with single-hole and high-resistance (~6–7 MΩ) were used for CHO cell recordings. Voltage pulses and whole-cell recordings were achieved using the PatchControl384 v1.5.2 software (Nanion, Munich, Germany) and the Biomek v1.0 interface (Beckman Coulter). Prior recordings, dissociated cells were shaken at 60 RPM in a cell hotel reservoir at 10 °C. After cell catching, sealing, whole-cell formation, liquid application, recording, and data acquisition were all performed sequentially and automatically. The intracellular solution contained (in mM): 10 CsCl, 110 CsF, 10 NaCl, 1 MgCl_2_, 1 CaCl_2_, 10 EGTA and 10 HEPES (pH 7.2, osmolarity 280 mOsm), and the extracellular solution (in mM): 140 NaCl, 4 KCl, 2 CaCl_2_, 1 MgCl_2_, 5 Glucose and 10 HEPES (pH 7.4, osmolarity 298 mOsm). Whole-cell experiments were done at −100 mV holding potential and at room temperature (18–22 °C), while currents triggered at −10 mV test potential were sampled at 20 kHz. Stimulation frequency was set at 0.2 Hz. Toxins were prepared at various concentrations in the extracellular solution, itself supplemented with 0.3% BSA. The peptides were distributed in 384-well compound plates according to the number of toxins to be tested (generally four), the concentration range tested according to the assumed IC_50_ values, and the number of cells desired for each experimental condition. Compound solutions were diluted 3 times in the patch-clamp recording well by adding 30 μL to 60 μL external solution to reach the final reported concentration and the test volume of 90 μL. Percentages of current inhibitions were measured at the end of a 12- to 15-min application time (12-min for fast blocking toxins; 15-min for slow blocking ones). A single concentration of peptide was tested on each cell for building the full-inhibition curves.

### 5.6. Formalin Pain Test

Pain behavior experiments were performed in C57BL/6J mice with the formalin test which evaluates behavioral responses to subcutaneous injection of 10 µL of 5% formalin into the plantar surface of the right hindpaw. The total time spent in licking and biting the right hindpaw over the next 45 min and divided into two phases (acute phase I, from 0 to 10 min, and inflammatory phase II, from 10 to 45 min) was determined and used as “pain” parameter. The effects of drugs on acute and inflammatory pain were evaluated after intrathecal injection of PhlTx1 (10 µL at 10 µM), or vehicle (NaCl 0.9%) (10 µL) or morphine-HCl (Cooper, 10 µL: 0.25 mg). Data were analyzed with GraphPad Prism 4. After testing the normality of data distribution, the statistical difference between different groups was analyzed using one way Anova followed by a Newman-Keuls multiple comparison test when *p* < 0.05. Mice procedures were approved by the Institutional Local Ethical Committee and authorized by the French Ministry of Research according to the European Union regulations and the Directive 2010/63/EU (Agreements C061525, NCE/2011-06 and 01550.03).

## Figures and Tables

**Table 1:** Comparison of IC_50_ values (in nM) for the human Na_v_1.7 channel of toxins studied here or published (Publ.) as referenced (Ref.). Published data for GrTx1, GsAFI and GsAFII arise from purified peptides.

Family	NaSpTx1					NaSpTx2		NaSpTx3			
Toxin	PhlTx1	HnTx-III	HnTx-IV	HwTx-I	HwTx-IV	ProTx-I	Pn3a	GrTx1	ProTx-II	GsAFI	GsAFII
IC_50_	39	50.6	4.3	25.1	9.6	7.1	1457	15.3	1.5	1.0	13.6
Publ.	250	232	21	630	26	72	0.9	370	1	40	1030
Ref.	[47]	[51]	[52]	[55]	[50]	[56]	[45]	[54]	[57]	[54]	[54]
